# Low Evolutionary Selection Pressure in Senescence Does Not Explain the Persistence of Aβ in the Vertebrate Genome

**DOI:** 10.3389/fnagi.2019.00070

**Published:** 2019-03-28

**Authors:** Robert D. Moir, Rudolph E. Tanzi

**Affiliations:** Genetics and Aging Research Unit, Department of Neurology, MassGeneral Institute for Neurodegenerative Disease, Harvard Medical School – Massachusetts General Hospital, Boston, MA, United States

**Keywords:** Alzheimer’s disease, amyloid-β protein, senescence, antimicrobial peptide, species fitness, menopause, selection pressure

## Abstract

The argument is frequently made that the amyloid-β protein (Aβ) persists in the human genome because Alzheimer’s disease (AD) primarily afflicts individuals over reproductive age and, therefore, there is low selective pressure for the peptide’s elimination or modification. This argument is an important premise for AD amyloidosis models and therapeutic strategies that characterize Aβ as a functionless and intrinsically pathological protein. Here, we review if evolutionary theory and data on the genetics and biology of Aβ are consistent with low selective pressure for the peptide’s expression in senescence. Aβ is an ancient neuropeptide expressed across vertebrates. Consistent with unusually high evolutionary selection constraint, the human Aβ sequence is shared by a majority of vertebrate species and has been conserved across at least 400 million years. Unlike humans, the overwhelming majority of vertebrate species do not cease reproduction in senescence and selection pressure is maintained into old age. Hence, low selective pressure in senescence does not explain the persistence of Aβ across the vertebrate genome. The “Grandmother hypothesis” (GMH) is the prevailing model explaining the unusual extended postfertile period of humans. In the GMH, high risk associated with birthing in old age has lead to early cessation of reproduction and a shift to intergenerational care of descendants. The rechanneling of resources to grandchildren by postreproductive individuals increases reproductive success of descendants. In the GMH model, selection pressure does not end following menopause. Thus, evolutionary models and phylogenetic data are not consistent with the absence of reproductive selection pressure for Aβ among aged vertebrates, including humans. Our analysis suggests an alternative evolutionary model for the persistence of Aβ in the vertebrate genome. Aβ has recently been identified as an antimicrobial effector molecule of innate immunity. High conservation across the Chordata phylum is consistent with strong positive selection pressure driving human Aβ’s remarkable evolutionary longevity. Ancient origins and widespread conservation suggest the human Aβ sequence is highly optimized for its immune role. We detail our analysis and discuss how the emerging “Antimicrobial Protection Hypothesis” of AD may provide insights into possible evolutionary roles for Aβ in infection, aging, and disease etiology.

The hallmark pathology for Alzheimer’s disease (AD) is deposition of amyloid-β protein (Aβ) as β-amyloid senile plaques. Accumulation of high β-amyloid burden is thought to drive a succession of pathologies leading to neurodegeneration and dementia. This model is called the “Amyloid Cascade Hypothesis” (ACH) of AD. β-amyloid is generated in brain by the ordered self-assembly of Aβ into fibrils containing monomer units arranged as β-pleated sheets. Aβ fibrillization is widely viewed as an intrinsically abnormal and exclusively pathological activity. The Aβ peptide itself is most often characterized as a functionless incidental product of catabolism. However, evolutionary theory predicts negative reproductive selection pressure would rapidly eliminate a non-functional and highly pathogenic gene from the genome (Crow and Kimura, 1970). A “Low Selection Pressure in Senescence” (LSPS) argument has frequently been proposed to explain Aβ’s puzzling persistence in the human genome despite the peptide’s supposed lack of a physiological function and intrinsic pathogenicity. In the LSPS model, Aβ is not purged from the human genome because AD primarily afflicts individuals over reproductive age and, therefore, there is low reproductive selective pressure for the peptide’s elimination or modification. For over two decades the LSPS argument has provided support for amyloidogenesis models that ascribe amyloid generation in AD to an intrinsically abnormal propensity of Aβ for unconstrained self-association. Here, we present the first detailed evaluation of the LSPS argument. Our analysis shows the LSPS model is not consistent with modern evolutionary theory or data on the activities and genetics of Aβ. Our findings suggest Aβ persists across the vertebrate genome, not because of low reproductive selection pressure, but because the peptide increases inclusive fitness. Our analysis adds to mounting evidence suggesting an urgent need for revaluation of prevailing AD amyloidogenesis and therapeutic models that characterize Aβ as a functional less disease-causing catabolic by-product (reviewed by Moir et al., 2018). We examine how the new “Antimicrobial Protection Hypothesis” of AD (Moir et al., 2018) provides a fresh interpretation of the ACH that is consistent with preservation of Aβ in the vertebrate genome and the emerging role of innate immunity in AD etiology.

Human reproductive senescence occurs much faster than somatic aging and woman exhibit prolonged postreproductive periods that can extend to more than 30 % of normal lifespan (Peacock and Ketvertis, 2018). AD primarily afflicts individuals in this postreproductive period. In the LSPS model, genes mediating disease in humans are not subject to selection pressure postreproduction. However, this model does not address Aβ’s persistence in non-human genomes. The Aβ sequence is ancient and highly conserved across vertebrates (Figure 1; Luna et al., 2013). Recent findings suggest non-human vertebrates also suffer excessive β-amyloid deposition and Alzheimer’s-like cognitive impairment in senescence (Nakayama et al., 1999; Maldonado et al., 2002a,b; Youssef et al., 2016). However, postreproductive periods for the majority of iteroparous vertebrate species, if present at all, are less than 5% of normal lifespan and individuals continue to have offspring well into senescence (Jones et al., 2014; Croft et al., 2015; Field and Bonsall, 2017). For some vertebrae species, reproductive success is highest among older mothers (Reiter et al., 1981; Palumbi, 2004; Hixon et al., 2014). Thus, for most vertebrate species reproductive selection pressure does not cease in old age when pathologies associated with Aβ expression manifest. Nonetheless, the human Aβ sequence is shared by 60–70 % of vertebrates and has been conserved across at least 400 million years (Luna et al., 2013). Aged individuals also indirectly contribute to the reproductive success of kin in several ways (Roach and Carey, 2014). Among social mammals, the presence of aged mothers increases the reproductive success of daughters (Fairbanks and McGuire, 1986; Lahdenpera et al., 2016; Lee et al., 2016). The extensive habitat knowledge accumulated by old individuals is also an important part of the survival strategy of mammals living in close kin groups (McComb et al., 2001; Modlmeier et al., 2014). Evolutionary theory predicts functionless or harmful genes that reduce the support old mothers provide for reproduction among kin will be selected out of species genomes. However, Aβ remains widely expressed with the human sequence greater than 95 % conserved across mammals (Tharp and Sarkar, 2013). Thus, the LSPS model fails to explain Aβ’s remarkable evolutionary persistence among non-human vertebrates.

**FIGURE 1:**
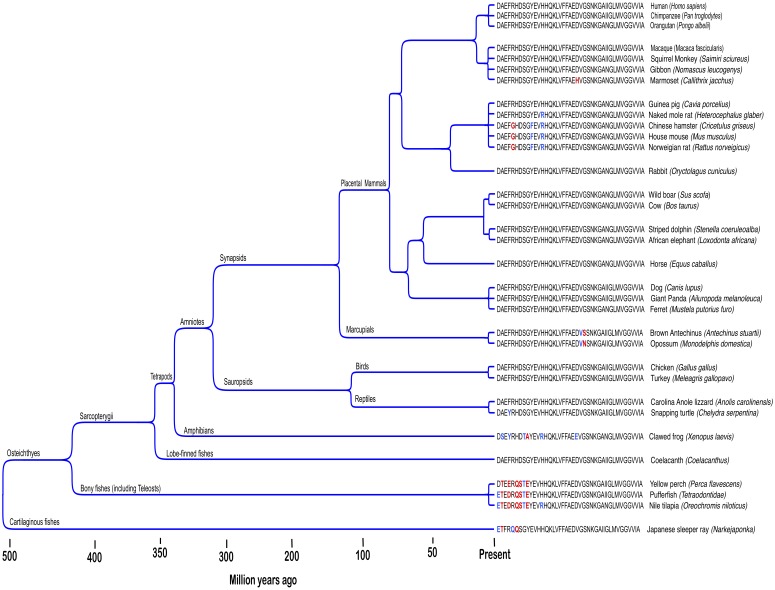
Amyloid-β protein is an ancient and highly evolutionary conserved neuropepetide. Figure shows Aβ42 sequences across different vertebrate taxa. Conserved (blue) and non-conserved (red) amino acid substitutions among non-human species are highlighted. Adapted from Luna et al. (2013).

In the LSPS argument, reproductive selection pressure is low for humans following the end of reproduction. To date, only humans, killer whales (*Orcinus orca*) and short-finned pilot whales (*Globicephala macrorhynchus*) have been found to undergo menopause (Marsh and Kasuya, 1986; Olesiuk et al., 1990). Menopause is thought to have evolved independently in humans and toothed cetaceans because of important lifestyle traits these species share, including highly social behaviors focused around family groups with elevated local relatedness, a long adolescence during which offspring learn diverse survival skills and acquire local and often highly specialized knowledge from their mothers and kin, and high maternal risk associated with birthing in old age. Ongoing debate continues to refine evolutionary theory on the origin of menopause (Hawkes and Coxworth, 2013; Croft et al., 2015). However, a broad consensus has emerged that postreproductive lifespan in these species increases inclusive fitness. The most widely accepted theory is known as the “Grandmother Hypothesis” (GMH) and explains menopause as an adaptation mediating extended kin networking in species for which reproduction in senescence carries high risk (Williams, 1957). In this model, postreproductive individuals rechannel reproductive energy and resources (including experience and knowledge) to grandoffspring. This provision of intergenerational care promotes survival of descendants and increases species evolutionary fitness. The GMH model has been confirmed in whales where postfertile females play an important role in increasing survival and reproductive success of descendants and close relatives (Foster et al., 2012; Brent et al., 2015; Croft et al., 2017). Delineating the benefits of grandparenting in human societies has proved more challenging. However, mounting data from both modern and less technological advanced societies are consistent with reproductive benefits for family groups that include grandparents (Hawkes, 2003; Ragsdale, 2004; Shanley et al., 2007; Pavard et al., 2008; Lahdenpera et al., 2012; Mace and Alvergne, 2012; Cyrus and Lee, 2013; Hooper et al., 2015). Thus, inclusive fitness theory (Kirkwood and Shanley, 2010) and data on human reproduction are not consistent with low evolutionary selection pressure for postreproductive individuals as posited in the LSPS model. Rather, persistence of Aβ in the human and vertebrate genomes suggests strong positive selection pressures are driving the protein’s enduring and widespread evolutionary conservation.

Aβ is generated by proteolytic cleavage of the amyloid-β precursor protein (APP). Aβ generation requires the peptide’s excision from APP by β-secretase (β-site APP cleaving enzyme [BACE1]) and a γ–secretase complex. Aβ generation has been confirmed in a range of sarcopterygians. Findings for zebrafish (*Danio rerio*) and kokanee salmon (*Oncorhynchus nerka kennerlyi*) are also consistent with generation of Aβ by teleosts (Maldonado et al., 2000, 2002a,b; Nada et al., 2016; Nery et al., 2017; Pu et al., 2017), despite analysis suggesting fish APP lacks the classical BACE1 cleavage site found in other vertebrates (Moore et al., 2014). Thus, most vertebrates appear to actively generate Aβ from APP. Phylogenetic analysis indicates the ancestral APP/Aβ gene arose with metazoic speciation during the Ediacaran period (Tharp and Sarkar, 2013). Early gene duplication lead to a family of three homologs in vertebrate species: APP, amyloid precursor-like proteins 1 (APLP1), and amyloid precursor-like proteins 2 (APLP2) (Wasco et al., 1992, 1993). The Aβ sequence is the most highly conserved domain within the vertebrate APP family (Tharp and Sarkar, 2013). APLP1 and APLP2 contain homologous Aβ domains that are less evolutionarily conserved, varying between each other and across species (Tharp and Sarkar, 2013). The unique conservation of the Aβ domain is consistent with an ancient and important physiological role for this peptide sequence. Aβ is part of the APP transmembrane domain. However, data suggest the apparent high evolutionary selection constraint of human Aβ is not mediated by the domains role as part of the APP holoprotein. The Aβ homolog regions in APLP1 and APLP2 are distinct from the Aβ domain in APP. Data from genetically modified cell and animal models confirm that APP, APLP1, and APLP2 share activities and have partially overlapping functions (Muller et al., 2017). In addition, murine APP contains a non-human Aβ sequence but the protein appears to retain full functionality (Deyts et al., 2016; Kaneshiro et al., 2018). These data suggest the human Aβ sequence is sufficient, but not essential, for functionality of the members of the APP protein family. Thus, the evolutionary conservation of the Aβ sequence is most likely linked to the actions of the excised peptide rather than activities of the APP holoprotein. Rates of protein sequence evolution depend primarily on the level of functional constraint (Zhang and Yang, 2015). Protein evolution models predict that optimized genes important for species fitness show high sequence stability over large evolutionary periods (Zhang and Yang, 2015). Proteins subject to low selection pressure accumulate mutations and display genetic drift across species (Boucher et al., 2014). Indeed, genetic drift mediated by low selection pressure is thought to be key for the generation of novel proteins (Boucher et al., 2014). Hence, from an evolutionary perspective, high sequence conservation and persistence among vertebrates across at least 400 million years is consistent with a strong association between Aβ expression and increased species fitness. Moreover, widespread preservation of the human Aβ sequence among vertebrates (Luna et al., 2013) does not support AD amyloidosis models that characterize fibrillization as intrinsically abnormal. In contrast to prevailing AD amyloidogenesis models, intergenetic data suggest Aβ fibrillization is associated with high evolutionary selective constraint, consistent with an important beneficial role for β-amyloid generation in non-AD brain. However, until recently it has been unclear what physiological role Aβ fibrillization normally plays.

We (Soscia et al., 2010; Kumar et al., 2016; Eimer et al., 2018), and other independent laboratories (White et al., 2014; Bourgade et al., 2015, 2016; Spitzer et al., 2016), recently identified Aβ as an antimicrobial peptide (AMP). AMPs are the primary effector proteins of the innate immune system. The microbial inhibitory activities of AMPs are critically important for host immunity and they target bacteria, mycobacteria, enveloped viruses, fungi, protozoans, and, in some cases, transformed or cancerous host cells (Wiesner and Vilcinskas, 2010). However, AMP activities are not limited to antibiotic-like actions. AMPs often play multiple diverse roles in immunity. To greater or lesser extents, all of the roles Aβ plays as an AMP are likely to influence the peptide’s evolutionary conservation. Germaine to AD, AMPs are potent immunomodulators (Steinstraesser et al., 2011) and are sometimes called the alarmins because of their cytokine-like proinflammatory activities. Consistent with identity as an AMP, synthetic Aβ inhibits fungal, bacterial, and viral pathogens *in vitro* (Soscia et al., 2010; White et al., 2014; Bourgade et al., 2015, 2016; Spitzer et al., 2016). Most recently, we have shown human Aβ expression *in vivo* protects against pathogens in transformed 3D human neuronal cell culture and transgenic *C. elegans* and AD mouse infection models, doubling host survival in some cases (Kumar et al., 2016; Eimer et al., 2018). Conversely, genetically modified mice lacking APP or the secretases required for Aβ generation, show attenuated infection resistance (Dominguez et al., 2005; Kumar et al., 2016; Eimer et al., 2018). Amyloid fibrils mediate the direct microbe inhibitory activities of Aβ. Aβ oligomers first bind carbohydrate moieties on microbial surfaces. Bound oligomers then provide a nidus and anchor for Aβ fibril propagation. Growing Aβ fibrils capture, agglutinate, and finally entrap microbes in a protease-resistant network of β-amyloid. In the antimicrobial Aβ fibrilization model, seeding of β-amyloid by pathogenic microorganisms is part of a protective innate immune response to infection. In AD, sustained activation of this pathway leads to amyloidosis and pathology. However, Aβ fibrilization and amyloid generation *per se* are not abnormal and mediate a protective immune pathway. This newly identified role for β-amyloid is consistent with our sequence evolution analysis that suggests Aβ fibrilization mediates beneficial immune functions. Also consistent with this emerging view of Aβ is the role peptide fibrillization plays in mediating the protective antimicrobial actions of classical AMPs, including lytic (Radzishevsky et al., 2008; Sood et al., 2008; Chu et al., 2012; Kagan et al., 2012) and agglutination/entrapment (Tsai et al., 2011; Chu et al., 2012; Torrent et al., 2012) activities.

Findings from our phylogenetic analyses are consistent with emerging data showing a role for Aβ fibrillization pathways in innate immunity. This stands in stark contrast to prevailing models that characterize fibrilization and associated Aβ activities as intrinsically abnormal. The view that Aβ activities are abnormal arose from an early surmise about the peptide’s origins that, while plausible at the time, has since proved inaccurate. Three and a half decades ago when Aβ generation was first characterized, intramembrane protein cleavage was viewed as an abnormal and exclusively disease-associated pathway (Kang et al., 1987). APP intramembrane cleavage and Aβ generation were thought limited to AD brain (Sisodia et al., 1990). As an abnormal catabolic product generated only under disease conditions, Aβ was presumed to lack a normal physiological function. However, intramembrane cleavage is now recognized as a normal proteolytic pathway mediating generation of diverse functional biomolecules (Rawson et al., 1997). Furthermore, findings have confirmed Aβ is a widely and constitutively expressed vertebrate neuropeptide (Figure 1; Luna et al., 2013; Tharp and Sarkar, 2013). However, while early assumptions about Aβ’s origin proved incorrect, the amyloidogenesis models they helped engender remain widely held. Moreover, the LSPS hypothesis continues to be cited in support of these longstanding amyloidogenesis models. However, as our analysis underscores, data accumulated over the last three decades is inconsistent, not only with early speculations on Aβ’s origin, but also the longstanding LSPS argument.

Data are consistent with lifelong positive selection pressure mediating conservation and persistence of Aβ in the vertebrate genome. However, antagonistic pleiotropy may also play a role in the etiology of patients with high genetic risk for AD. In the antagonistic pleiotropy hypothesis, a gene beneficial to evolutionary fitness early in life may be detrimental in senescence- early benefits outweighing later costs (Williams, 1957). The ε4 allele (*APOE4*) of the apolipoprotein E gene is associated with enhanced β-amyloid deposition and increased AD risk (Strittmatter et al., 1993). An antagonistic pleiotropy model has been proposed for the pathogenicity of *APOE4* in inflammation-associated late-life diseases, including AD (Jasienska et al., 2015), arteriosclerosis (Mahley, 1988), multiple sclerosis (Chapman et al., 2001), ischemic cerebrovascular disease (McCarron et al., 1999), sleep apnea (Kadotani et al., 2001), and pathologies resulting from traumatic brain injury (Friedman et al., 1999). *APOE* is important for immunity and genetically modified mice lacking the protein show attenuated pathogen resistance (Miller and Federoff, 2008). All three human apoE isoforms (apoE2, apoE3, and apoE4) modulate immunity but *APOE4* carriers appear to have heightened immune responsiveness (Vitek et al., 2009; Gale et al., 2014; van Exel et al., 2017; Nam et al., 2018). The augmented innate immune response associated with apoE4 expression is thought to exacerbate inflammation-mediated pathologies (van Exel et al., 2017; Corbett et al., 2018). However, in high pathogen environments expression of apoE4 is associated with increased fertility and juvenile survival compared to *APOE2* or *APOE3* carriers (Oria et al., 2010; Vasunilashorn et al., 2011; Mitter et al., 2012; Fujioka et al., 2013; Trumble et al., 2017; van Exel et al., 2017). Inheritance of *APOE4* is also associated with improved cognitive function among populations with high parasite burdens (Azevedo et al., 2014). Thus, a “hair-trigger” immune response for *APOE4* carriers may protect against infection early in life. With regard to *APOE4’s* involvement in AD, an antagonistic pleiotropy model is consistent with the recently emerged innate immune role for Aβ fibrillization pathways. In an AD antagonistic pleiotropy model, increased proclivity for β-amyloid generation may be beneficial in young individuals, providing *APOE4* carriers with a more robust protective response to neuroinfection. However, apoE4-enhanced Aβ fibrillization may also promote amyloidosis, leading to harmful AD pathology in late-life.

An antagonistic pleiotropy model for the role of apoE in AD amyloidosis is consistent with etiology data and evolutionary explanations for the protein’s involvement across multiple age-dependent inflammation diseases (Corbett et al., 2018). However, it is less clear if Aβ fibrillization itself should be considered as antagonistic pleiotropy independent of *APOE4*. Three decades of accumulated data link AD etiology to increased microbial burden in brain. Recent findings on the protective immune entrapment role of Aβ suggest elevated brain microbe levels may mediate AD amyloidosis. If β-amyloid is helping protect AD patients from chronic and potentially lethal neuroinfection, then amyloidosis is playing a beneficial immune role in late life and Aβ fibrillization activities do not satisfy classical criteria for antagonistic pleiotropy. Rather, this suggests a model in which β-amyloid deposition is an early innate immune response to persistent immunochallenge. We call this the “Antimicrobial Protection Hypothesis” of AD (Moir et al., 2018). Amyloid generation in the antimicrobial protection model is an immune defense pathway that entraps pathogens. Aβ fibrils generated to trap microbes also drive neuroinflammatory pathways that help fight the infection and clear β-amyloid/pathogen deposits. In AD, chronic activation of this pathway (caused by genuine infection or an incorrectly perceived immunochallenge) helps drive the tauopathy and sustained neuroinflammation pathologies that lead to neurodegeneration and dementia. This model is consistent with the ACH in which amyloid deposition drives a succession of pathologies that end in dementia. However, in this model amyloidosis is not drive by an intrinsically harmful and functionless propensity of Aβ to self-associate as in prevailing models. The potential for pathological outcomes from Aβ activities is consistent with the protective/harmful duality shown for classical AMPs and innate immune responses across multiple diseases (Shastri et al., 2013). Furthermore, genetic data on the role of rare mutations in FAD are also consistent with the antimicrobial protection model. FAD mutations shift Aβ isoform ratios, leading to amyloidosis (Tanzi, 2012). Mutation-mediated changes in isoform expression among classical AMPs also mediate disease pathology. For example, inherited mutations that shift human β-defensin 1 isoform ratios enhance atopic disorders, including asthma (Cagliani et al., 2008). Enhanced amyloidosis associated with FAD mutations parallel the mutation-induced upregulation of innate immune pathways that mediate pathologies in inherited autoinflammatory syndromes, including Familial Mediterranean fever, TNF receptor- associated periodic syndrome, Muckle–Wells syndrome, Blau syndrome, pyogenic arthritis, pyoderma gangrenosum and acne syndrome, early-onset enterocolitis, autoinflammation and PLCγ2- associated antibody deficiency and immune dysregulation, and proteasome-associated autoinflammatory syndromes (Martinon and Aksentijevich, 2015). The Antimicrobial Protection Hypothesis provides a framework for rational incorporation of the genetics and seemingly disparate pathologies involved in AD neurodegeneration. The new model remains broadly consistent with the ACH of AD. However, in the antimicrobial protection interpretation of ACH, the modality of Aβ’s pathological actions in AD is shifted from abnormal stochastic behavior toward sustained innate immune activity. Moreover, persistence of Aβ in the human genome is not mediated by LSPS, but by the peptide’s lifelong contribution to inclusive fitness.

A focus on Aβ fibrillization pathways advanced our understanding of amyloidosis early in the modern molecular/genetic era of AD research. Unfortunately, there have been few attempts in the intervening years to critically reevaluate longstanding amyloidosis models from this era in light of emerging genetic and molecular data. The LSPS argument is a conspicuous example of how seemingly plausible, but ultimately deeply flawed models can persistence in the absence of continuing critical reevaluation. Amyloidosis driven by an intrinsically and exclusively pathological Aβ peptide has been the dominant AD pathogenic model for over three decades. This characterization of Aβ has lead to an intense focus on strategies aimed at limiting or eliminating the peptide. However, to date, this therapeutic approach has been singularly unsuccessful. Prevailing Aβ pathogenesis models are reminiscent of the parable about the elephant and three self-proclaimed wise men. Each blindfolded man touched a different part of an elephant and loudly proclaimed three different, and equally wrong, assertions as to the beast’s nature. Aβ activities are typically considered as discrete abnormal pathways and variously ascribed pathological roles in AD. A possible overarching physiological function for the collective activities of Aβ has rarely been considered. We believe the Antimicrobial Protection Hypothesis can provide a rational framework for incorporating seemingly independent findings on Aβ and help advance a new understanding of AD amyloidogenesis. We also believe a fuller appreciation of the ancient origin and important role Aβ fibrillization plays in immunity will prove important for the future development of effective AD treatment strategies.

## Author Contributions

RM and RT contributed equally to the preparation and analysis presented in this manuscript.

## Conflict of Interest Statement

The authors declare that the research was conducted in the absence of any commercial or financial relationships that could be construed as a potential conflict of interest.
